# A Novel *COL4A5* Mutation Identified in a Chinese Han Family Using Exome Sequencing

**DOI:** 10.1155/2014/186048

**Published:** 2014-07-06

**Authors:** Xiaofei Xiu, Jinzhong Yuan, Xiong Deng, Jingjing Xiao, Hongbo Xu, Zhaoyang Zeng, Liping Guan, Fengping Xu, Sheng Deng

**Affiliations:** ^1^Center for Experimental Medicine and Department of Neurology, The Third Xiangya Hospital, Central South University, Changsha, Hunan 410013, China; ^2^Department of Pharmacy, Xiangya Hospital, Central South University, Xiangya Road 87, Kaifu District, Changsha, Hunan 410008, China; ^3^Department of Nephrology, The Third Xiangya Hospital, Central South University, Changsha, Hunan 410013, China; ^4^BGI-Shenzhen, Shenzhen, Guangdong 518083, China; ^5^Cancer Research Institute, Xiangya Medical School of Central South University, Changsha, Hunan 410008, China

## Abstract

Alport syndrome (AS) is a monogenic disease of the basement membrane (BM), resulting in progressive renal failure due to glomerulonephropathy, variable sensorineural hearing loss, and ocular anomalies. It is caused by mutations in the collagen type IV alpha-3 gene (*COL4A3*), the collagen type IV alpha-4 gene (*COL4A4*), and the collagen type IV alpha-5 gene (*COL4A5*), which encodes type IV collagen *α*3, *α*4, and *α*5 chains, respectively. To explore the disease-related gene in a four-generation Chinese Han pedigree of AS, exome sequencing was conducted on the proband, and a novel deletion mutation c.499delC (p.Pro167Glnfs*36) in the *COL4A5* gene was identified. This mutation, absent in 1,000 genomes project, HapMap, dbSNP132, YH1 databases, and 100 normal controls, cosegregated with patients in the family. Neither sensorineural hearing loss nor typical *COL4A5*-related ocular abnormalities (dot-and-fleck retinopathy, anterior lenticonus, and the rare posterior polymorphous corneal dystrophy) were present in patients of this family. The phenotypes of patients in this AS family were characterized by early onset-age and rapidly developing into end-stage renal disease (ESRD). Our discovery broadens the mutation spectrum in the *COL4A5* gene associated with AS, which may also shed new light on genetic counseling for AS.

## 1. Introduction

Alport syndrome (AS) is a monogenic disease of the basement membrane (BM), resulting in progressive renal failure due to glomerulonephropathy, variable sensorineural hearing loss, and ocular anomalies. It is caused by defects of type IV collagen, which is the major structural component of BM and necessary for BM maintenance [1]. Type IV collagen comprises six *α* chains (*α*1–*α*6) encoded by the collagen type IV alpha-1 gene (*COL4A1*) to the collagen type IV alpha-6 gene (*COL4A6*), respectively. These six *α* chains share a common primary structure: an approximately 25-residue “7S” domain at the amino terminus, a collagenous domain of approximately 1,400 Gly-X-Y repeats, and an approximately 230-residue noncollagenous (NC1) domain at the carboxyl terminus [2]. AS is caused by mutations in the collagen type IV alpha-3 gene (*COL4A3*), the collagen type IV alpha-4 gene (*COL4A4*), and the collagen type IV alpha-5 gene (*COL4A5*), encoding type IV collagen *α*3, *α*4, and *α*5 chains, respectively [3]. The estimated gene mutation frequency is 1/5,000–1/10,000 [4]. Three inheritance patterns of AS have been reported: the most common X-linked inheritance (mutations in the* COL4A5* gene; ~85%), the less common autosomal recessive inheritance (mutations in the* COL4A3* gene and the* COL4A4* gene; ~15%), and the rare autosomal dominant inheritance [5]. Genotype-phenotype correlations of AS have been extensively described. Patients may present with a wide spectrum of phenotypes, ranging from benign familial hematuria (BFH) or thin basement membrane nephropathy (TBMN) to end-stage renal disease (ESRD) resulting from various mutations, though the* COL4A5*-related BFH and TBMN were considered to be the milder subtypes of AS [5–7].

The purpose of our study is to explore the disease-related gene in a four-generation Chinese Han pedigree of AS. Exome sequencing is a powerful and cost-effective tool for uncovering the genetic basis of diseases [8, 9]. Conventional mutation screening by Sanger sequencing is time consuming and expensive due to genetic heterogeneity of AS and large size of those three genes (*COL4A3*,* COL4A4*, and* COL4A5*). Therefore, we detected the proband of the family using exome sequencing to identify the gene responsible for this disease. A novel deletion mutation c.499delC (p.Pro167Glnfs*36) in the* COL4A5* gene was identified, and it cosegregated with the disease in the family. Our data broaden the genotypic spectrum of* COL4A5* mutations associated with AS.

## 2. Materials and Methods

### 2.1. Subjects

**Figure 1:**
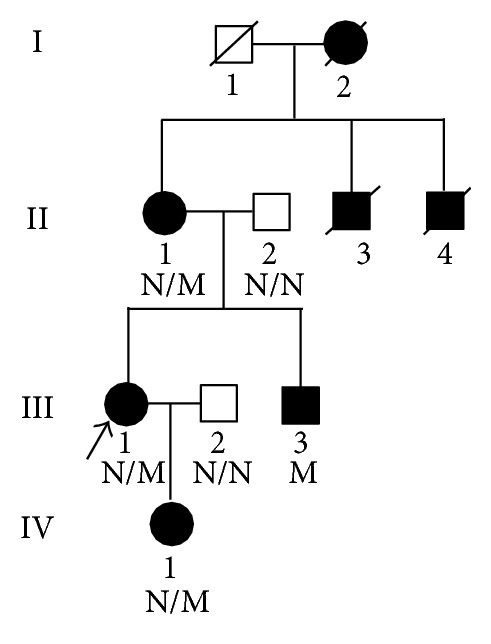
Pedigree of the family with X-linked Alport syndrome. N: normal, M:* COL4A5* c.499delC (p.Pro167Glnfs*36) mutation.

A pedigree consisting of 10 individuals across 4 generations of Chinese Han family was enrolled in this study (Figure 1). Peripheral blood samples were collected from 6 members of this family, including 4 patients. Peripheral blood samples were also collected from 100 unrelated ethnically matched normal controls (male/female: 50/50, age 40.6 ± 8.4 years). All participants underwent clinical evaluation, auditory and typical* COL4A5*-related ophthalmological examinations (dot-and-fleck retinopathy, anterior lenticonus, and the rare posterior polymorphous corneal dystrophy). The protocol of this study was approved by the Ethics Committee of the Third Xiangya Hospital, Central South University, and all participants signed informed consent.

### 2.2. Clinical Data

All family members underwent urinalysis and renal function evaluation. Members with no more than trace amount of hematuria or proteinuria and normal renal ultrasound examination were considered normal [10]. Kidney biopsy was performed for the proband. Global and segmental sclerosis and mesangial expansion were identified by light microscopy. Electron microscopy revealed irregular thickening and splitting of the glomerular basement membranes (GBMs). Immunofluorescence and electron microscopy detected no immunoglobulin A (Ig A) deposition. None of the family members showed any evidence of auditory, typical* COL4A5*-related ophthalmological (dot-and-fleck retinopathy, anterior lenticonus, and posterior polymorphous corneal dystrophy), or platelet abnormalities or leiomyomatosis.

### 2.3. Exome Capture

Genomic DNA was isolated from peripheral blood leukocytes by standard phenol-chloroform extraction method [11]. Three micrograms (*μ*g) of genomic DNA was used to construct the exome library. Genomic DNA of the proband was sheared by sonication and hybridized to the Nimblegen SeqCap EZ Library for enrichment, according to the manufacturer's protocol. Enriched exome fragments were sequenced on the HiSeq 2000 platform (Illumina, San Diego, CA, USA) to get paired-end reads with read length of 90 bp. A mean exome coverage of 81.65× was obtained to accurately call variants at 99.41% of the targeted exome [12, 13].

### 2.4. Read Mapping and Variant Analysis

The sequence reads were aligned to human genome reference obtained from UCSC database (http://genome.ucsc.edu/), version hg19 (build 37.1), using the program SOAP aligner. Single nucleotide polymorphisms (SNPs) were called using SOAPsnp set with the default parameters after the duplicated reads (produced mainly in the PCR step) were deleted [14]. Short insertions or deletions (indels) altering coding sequence or splicing sites were also identified by GATK. We filtered candidate SNPs with the following criterion: SNP quality ≥20, sequencing depth ≥4, the estimated copy number ≤2, and the distance between two SNPs >5 (the quality score is a Phred score, generated by the program SOAPsnp1.03, and quality score 20 represents 99% accuracy of a base call) [6]. Candidate mutations were filtered against databases including the single nucleotide polymorphism database (dbSNP132, http://www.ncbi.nlm.nih.gov/projects/SNP/snp_summary.cgi/), 1,000 genomes data (1,000 genomes release_20100804), HapMap (2010-08_phase II + III) and YanHuang1 (YH1) project, and synonymous substitutions. Potential disease-causing variants were evaluated by SIFT prediction (http://sift.jcvi.org/). Sanger sequencing was employed to verify the identified potential disease-causing variants with ABI3500 sequencer (Applied Biosystems, Foster City, CA, USA). Sequences of the primers were as follows: 5′-TGAATCTTCAGATCATTTTTCTGG-3′ and 5′-GAGGGATTGTTGTAATCTTCTGG-3′.

## 3. Results

We performed exome sequencing of the proband (III: 1, Figure 1) in a Chinese Han family with AS. We generated 8.14 billion bases of 90-bp paired-end read sequence for the patient. Among the 8.14 billion bases, 7.88 billion (96.81%) passed the quality assessment, 7.37 billion (93.53%) aligned to the human reference sequence, and 3.60 billion bases (48.85%) mapped to the targeted bases with a mean coverage of 81.65-fold. 105,963 genetic variants, including 14,723 nonsynonymous variants, were identified in either the coding regions or the splice sites. A prioritization scheme was applied to identify the pathogenic mutation in the patient, similar to recent studies [6, 15]. We excluded known variants identified in 1,000 genomes project, HapMap, dbSNP132, and YH1. Applying the above strategy, we reduced the number of candidate genes by more than 90.33%.

**Figure 2:**
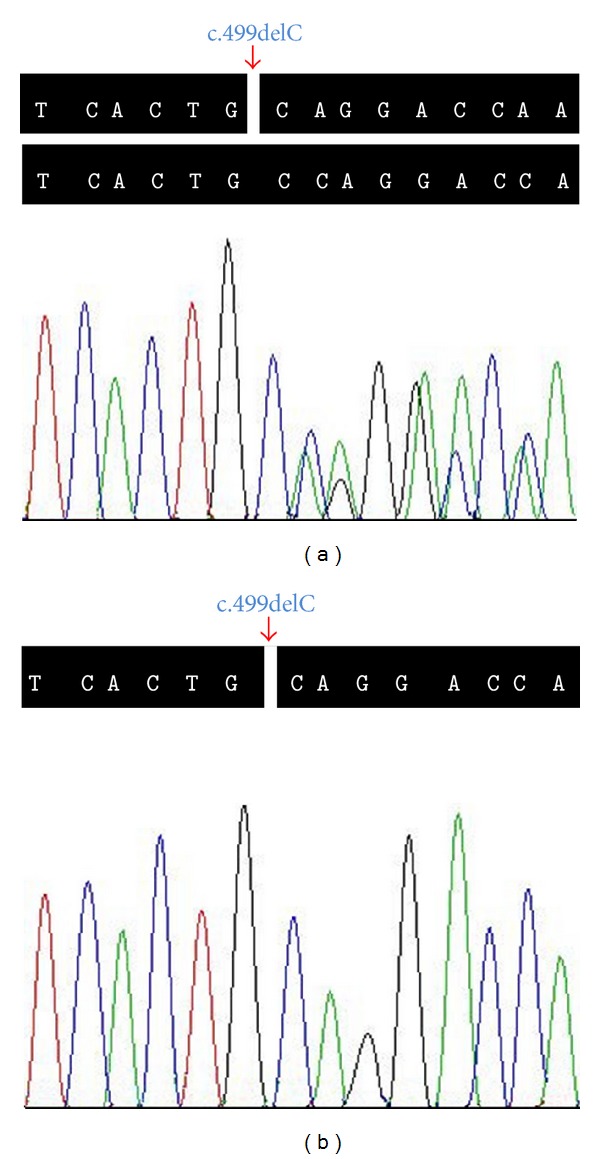
Sequencing analysis of* COL4A5* c.499delC (p.Pro167Glnfs*36) mutation. The arrow shows site of the novel c.499delC (p.Pro167Glnfs*36) deletion mutation in the* COL4A5* gene. (a) Heterozygous mutation carrier (III: 1). (b) Hemizygous mutation carrier (III: 3).

**Figure 3:**
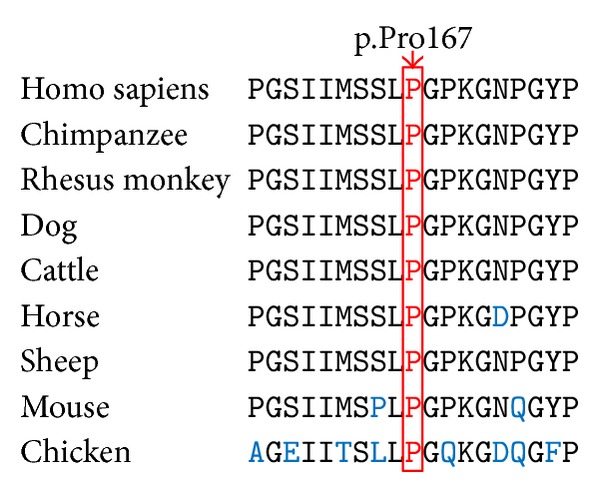
Conservation analysis of* COL4A5* p.Pro167 amino acid residue.

A novel deletion mutation, c.499delC (p.Pro167Glnfs*36), was identified in exon 9 of the* COL4A5* gene in the proband. This mutation results in premature stop codon and a truncated protein. The same mutation was subsequently verified in all four affected family members (II: 1, III: 1, III: 3, and IV: 1; Figure 1), while being absent in unaffected members and 100 ethnically matched normal controls by Sanger sequencing (Figure 2). It is also absent in 1,000 genomes project, HapMap, YanHuang1 (YH1) project, and dbSNP. The mutation is located in the Gly-X-Y repeats. The p.Pro167 is a highly conserved amino acid residue among different species from chicken to human, suggesting its structural and functional importance (Figure 3). This mutation was predicted to affect the protein features and be disease causing (predicted by http://www.mutationtaster.org/). SIFT prediction also showed a damaging effect with a confidence score of 0.858 (http://sift.bii.a-star.edu.sg/www/SIFT_indels2.html).

## 4. Discussion

AS is a clinically and genetically heterogeneous disease, and severity of this disease is usually equal between males and females in the autosomal recessive form (autosomal recessive AS, ARAS), while greater in males with X-linked form (X-linked AS, XLAS). XLAS is caused by mutations in the* COL4A5* gene with an approximately prevalence of 1/10,000 [5], and it accounts for 40%–45% of female patients with AS [16]. Female patients with XLAS have a variable and generally mild clinical course with 12% reaching ERSD by the age of 40 years and about 30% by the age of 60 years in European cohorts [17]. While male patients are more severe than females with 70% of affected males developing into ESRD before the age of 30 years (juvenile form), the remaining 30% are progressing toward ESRD after the age of 30 years (rare adult form) [18]. Furthermore, hearing loss and ocular abnormality happened in 90% and 35% of male patients, respectively [10].

In our family, four patients presented with heterogeneous clinical phenotypes of glomerulopathy, while none of them showed any clinical features of either sensorineural hearing loss or typical* COL4A5*-related ocular abnormalities. A* COL4A5* c.499delC (p.Pro167Glnfs*36) mutation in exon 9, cosegregating with the disease, was identified. The deletion mutation leads to a truncated protein and is absent in 1,000 genomes project, HapMap, YanHuang1 (YH1) project, dbSNP, and 100 normal controls. Our clinical and genetic data also support an X-linked inheritance form of AS in this family.

The* COL4A5* gene is located at Xq22 and contains 51 exons, encoding type IV collagen *α*5 chain [6]. Type IV collagen *α*5 chain contains 1,685 amino acid residues, which consist of a 26-residue signal peptide, a 1,430-residue collagenous domain starting with a 14-residue noncollagenous sequence, a Gly-X-Y-repeat sequence interrupted at 22 locations, and a 229-residue carboxyl-terminal NC1 domain [19]. To date, 688* COL4A5* mutations have been identified according to the Human Gene Mutation Database (http://www.hgmd.org/), including missense, nonsense, deletion, splicing mutation, and complex rearrangements [17, 20], without identification of any mutation hot spot. Genotype-phenotype correlations between* COL4A5* mutations and XLAS have been extensively described. For genotype-phenotype correlation purposes, typical XLAS is classified into three types: (1) severe type with ERSD at ~20 years (juvenile-onset ESRD), 80% of hearing loss, and 40% of ocular lesions, caused by large rearrangements, premature stop, frameshift, donor splice, and mutations in the NC1 domain; (2) moderate-severe type with ESRD at ~26 years, caused by non-Gly-X-Y-missense, Gly-X-Y mutations in 21–47 exons; (3) moderate type with ESRD at ~30 years (late-onset ESRD), 70% of hearing loss and <30% ocular lesions, caused by Gly-X-Y mutations in 1–20 exons [21, 22]. Four patients of our family showed no clinical features of either sensorineural hearing loss or typical* COL4A5*-related ocular abnormalities. Though our family is not large, a moderate type of XLAS is considered due to the mutation located in* COL4A5* exon 9 and the late-onset ESRD (ESRD at 36 years, II: 1; Table 1). More severe clinical phenotypes and earlier onset-age were observed in male patient of this family (III: 3), consistent with previous reports [6].

Mutations in genes encoding *α* chain of type IV collagen could lead to dysfunction of BM and then lead to the development of human disease in the eye, kidney, ear, and so forth [1]. Once the *α*5 chain is missing, the formation of the normal *α*3*α*4*α*5 (IV) protomer is disrupted in BM of glomerulus, ear, eye, and lung, which could lead to structural and functional defects [23]. This is supported by the immunohistochemical finding of frequent loss of *α*3, *α*4, and *α*5 signals in the GBM of XLAS patients [24, 25]. The cause of clinical heterogeneity of XLAS, such as difference in age of disease onset, disease severity, and disease progression, may be multifactorial, including random X chromosome inactivation, ethnic background, and environment factors.

Animal models with genetic deficiency may provide probabilities to reveal the pathogenesis and treatment of AS [26]. Two* Col4a5* truncation mutations have been identified in dogs (Samoyed and Navasota dogs) with clinical features of proteinuria and progressive kidney disease leading to terminal failure [27]. Intriguingly, a deletion in* Col4a5* resulting in disruption of the Gly-X-Y repeats, similar to human p.Pro167Glnfs*36 mutation, was observed in a family of mixed-breed dogs with an inherited nephropathy that exhibits the clinical, immunohistochemical, pathological, and ultrastructural features of human XLAS, and the truncated peptide chain may probably prevent extracellular assembly in type IV collagen networks [28]. Further studies on the* Col4a5* genetic-deficient AS animal models will provide new insight into mechanism research, diagnosis, and target therapy of AS in human.

## 5. Conclusions

In our study, we identified a novel deletion mutation c.499delC (p.Pro167Glnfs*36) in the* COL4A5* gene, which may be responsible for AS in this family. Our study showed that exome sequencing is a fast, sensitive, and relatively low-cost method to identify gene(s) responsible for AS. The discovery broadens the genotypic spectrum of* COL4A5* mutations associated with AS and has implications for genetic diagnosis, therapy, and genetic counseling of this family.

## Figures and Tables

**Table 1:** Clinical and genetic data of 4 *COL4A5* c.499delC (p.Pro167Gln*fs*∗36) mutation carriers.

Subject	II: 1	III: 1	III: 3	IV: 1
Gender	F	F	M	F
Age (year)	42	24	22	6
Onset-age (year)	13	20	3	3
Genotype	Heterozygote	Heterozygote	Hemizygote	Heterozygote
Renal function	ESRD at 36 years	Normal	Normal	Normal
Microscopic hematuria	Yes	Yes	Yes	Yes
Gross hematuria	Yes	No	Yes	No
Proteinuria	Yes	No	Yes	No
Uremia	Yes	No	No	No
Audiological examination	Normal	Normal	Normal	Normal
Ophthalmic examination^⋆^	No	No	No	No

^⋆^Dot-and-fleck retinopathy, anterior lenticonus and posterior polymorphous corneal dystrophy; *COL4A5*, the collagen type IV alpha-5 gene; F, female; M, male; ESRD, end-stage renal disease.
